# Microstructure Changes in Polyester Polyurethane upon Thermal and Humid Aging

**DOI:** 10.3390/polym8050197

**Published:** 2016-05-20

**Authors:** Qiang Tian, Ivan Krakovský, Guanyun Yan, Liangfei Bai, Jiahui Liu, Guangai Sun, László Rosta, Bo Chen, László Almásy

**Affiliations:** 1 Key Laboratory of Neutron Physics and Institute of Nuclear Physics and Chemistry, China Academy of Engineering Physics, Mianyang 621999, China; yanguany@126.com (G.Y.); bailiangfei@163.com (L.B.); guangaisun_80@163.com (G.S.); chenbo_58@163.com (B.C.); 2 Department of Macromolecular Physics, Faculty of Mathematics & Physics, Charles University, Prague 180 00, Czech Republic; ivan_krakovsky@yahoo.co.uk; 3 Institute of Chemical Materials, China Academy of Engineering Physics, Mianyang 621999, China; huiihuii@163.com; 4 Wigner Research Centre for Physics, Institute for Solid State Physics and Optics, P.O. Box 49, Budapest H-1525, Hungary; rosta.laszlo@wigner.mta.hu

**Keywords:** SANS, IR, polyurethane, aging, microstructure

## Abstract

The microstructure of compression molded Estane 5703 films exposed to 11%, 45%, and 80% relative humidity and 70 °C for 1 and 2 months has been studied by small-angle neutron scattering (SANS), Fourier transform infrared spectroscopy (FTIR), gel permeation chromatography (GPC), and differential scanning calorimetry (DSC). Scattering data indicated increase of the interdomain distance and domain size with a higher humidity and longer aging time. GPC data showed a progressive shortening of polyurethane chains with increasing humidity and aging time. The shortening of the polyurethane chains caused a drop of the glass transition temperature of soft segments, and promoted crystallization of the soft segments during long-time storage of the aged samples at room temperature. FTIR showed a substantial increase in the number of inter-urethane H-bonds in the aged samples. This correlates with the increase of the hard domain size and the degree of phase separation as measured by SANS. The data collected reveals that the reduced steric hindrance caused by hydrolysis of ester links in polybutylene adipate residues promotes the organization of hard segments into domains, leading to the increase of domain size and distance, as well as phase segregation in aged Estane. These findings provide insight into the effects of humidity and thermal aging on the microstructure of aged polyester urethane from molecular to nanoscale level.

## 1. Introduction

Thermoplastic polyurethanes (PUs) are a very attractive type of segmented polymers that bridge the gap between rubbers and plastics [1]. PUs have a wide range of industrial applications, and many of them are intended for long-term use. The mechanical stability of PUs depends on the structural changes induced by extended exposure to temperature, humidity, irradiation, and stress, occurring during fabrication, sterilization treatment, storage, and service. Understanding the microstructural changes during aging is necessary for the proper lifetime estimation and contributes to the general knowledge of structure-property relationships.

The system of interest in the present work is a kind of polyester based PU, Estane 5703 (hereafter called Estane), which has been widely used in adhesive for inks and lacquers, fabric coating, as well as magnetic media. Previous studies on the long term stability of Estane in thermal and humid environments revealed the destroying effect of humidity on the microstructure. As a common characteristic for PUs, the performance of Estane is not only determined by the chemical properties, but also strongly inﬂuenced by the microphase structure, in which the hard segments (HSs) segregate into domains (called *hard domains*), greatly increasing the modulus and strength by acting as rigid fillers. Early investigations on aged Estane mainly focused on chemical degradation and cross-linking [2,3,4,5]. Orler *et al.* [2] found that Estane aged in dry air quickly restored its initial mechanical properties, while the mechanical properties of the samples aged in wet air recovered only partially with time. The reason for this is that the ester soft segments (SSs) are more than an order of magnitude more susceptible to hydrolytic cleavage than the urethane [6]. This is an autocatalytic chain scission reaction that breaks an ester link to create a polymeric acid and a polymeric alcohol. Therefore, the high concentration of polyester SSs (77 wt %) in Estane leads to a higher probability of hydrolytic scission of macromolecular chains. Salazar *et al.* [4] identified that ester hydrolysis is the dominant mechanism of the molecular weight loss of Estane in thermal and humid environments.

On the other hand, up to now, only a few studies have pursued the behavior of the nanoscale morphology in aged Estane. Thompson *et al.* [7] studied the kinetics of phase separation in aged Estane. The samples were first aged at 70 °C with 75% and 0% relative humidity (RH), resulting in a decrease of molecular weight, then subjected to 100 °C to mix the polyester and the polyurethane domains, and finally cooled to room temperature and measured by infrared spectrometer as quickly as possible. By monitoring the relative intensities of vibrational changes, they inferred that the diffusion limited kinetics occurred at a much earlier time for the humid-aged samples with decreased molecular weight.

Small-angle scattering is a powerful tool for determination of the domain structure in polymers on the 1–50 nm scale, and can reveal structural changes reflected in variation of certain parameters such as domain size, interdomain distance, domain shape and orientation, and the degree of phase separation [8,9,10,11,12,13,14]. Mang [8] and Espada *et al.* [9] performed small-angle neutron scattering (SANS) measurements on a kind of explosive binder composed of Estane and plasticizer with ratio of 1 to 1. They found that the plasticizer is largely associated with the soft domains and stronger segregation between hard and soft domains as a result of the hydrolysis reaction. For these samples in which deuterated plasticizer was present, the obtained domain structure may be disturbed.

In summary, the aging process of Estane is complex and may involve more than one interrelated chemical and/or physical processes. The chemical and morphological processes must be considered for predicting the macroscopic properties and mechanical behavior. The chemical processes are chain scission or polymerization, which were extensive studied in the past. While the general aspects of phase change in polyurethane are known, the details of the morphological changes are far from being understood. We studied here the microstructure of aged Estane, aiming to clarify the following questions: (1) how the hard domain morphology and the degree of phase separation are affected by humid aging at elevated temperature; (2) what processes take place in the material on the molecular scale.

In this work, the microstructural changes in Estane aged in humid air with low, median, and high RH at elevated temperature is studied by various techniques. The domain morphology has been analyzed by SANS. In order to increase the contrast and data quality, the soft domains of Estane were swollen by deuterated solvent following a method proposed by Mang [15]. We have employed this method to study the structural changes of Estane aged in dry air and wet air [16]. More detailed information on morphological changes as a function of aging time and RH are obtained in the present study, and complemented by data on chain scission, hydrogen bonding, and thermal behavior obtained by gel permeation chromatography (GPC), Fourier transform infrared spectroscopy (FTIR), and differential scanning calorimetry (DSC) techniques. These studies contribute to the characterization of the aged structure in polyester PUs from a practical and industrial standpoint, and also clarifies the mechanism of morphological and chemical changes from a fundamental viewpoint.

## 2. Materials and Methods

### 2.1. Materials

**Figure 1:**
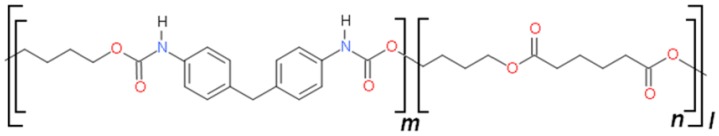
Chemical structure of the repeating units of Estane 5703. The part bracketed by *m* is the “hard segment,” the part bracketed by *n* is the “soft segment”. In Estane 5703: *m* ≈ 1–3 and *n* ≈ 4–6.

Estane 5703 was obtained as pellets from the Lubrizol Advanced Materials, Inc., Westerlo-Ovel, Belgium. It is a segmented polyurethane copolymer, containing about 23 wt % HSs, prepared by the reaction of 4,4’-diphenylmethane diisocyanate (MDI) and poly (butylene adipate) (PBA) with 1,4-butanediol (BDO) as the chain extender (Figure 1). It consists of HSs and SSs formed by MDI+2BDO and PBA residues, respectively. Estane pellets were compression-molded into 1 mm thick film at 20 MPa, 120 °C for 10 min and then cooled to room temperature (RT), initiating the phase separation process. The samples were kept in storage box at RT for one year before the aging experiments.

The films (15 mm × 8 mm × 1 mm) were sealed in containers to maintain the specified temperature and humidity environment. Aqueous saturated KCl, Mg(NO_3_)_2_, and LiCl solutions produced relative humidity (RH) of 80%, 45% and 11%, respectively. The containers were placed in convection ovens operating at 70 °C for 1 and 2 months. After treatment, the films were kept at RT in sample storage box with a constant humidity of 30% for not less than 6 months before the microstructural characterizations. During this time, the excess absorbed water was largely removed from the samples aged at medium and high humidity, and the phase separation of hard and soft domains took place. Estane films stored in the same storage box at RT without thermal and humid aging were taken as the reference samples.

### 2.2. Characterization

The SANS measurements were performed with the small-angle neutron scattering diffractometer *Yellow Submarine* at the Budapest Research Reactor. The scattering intensity *I*(*q*) is measured as a function of scattering vector *q* = 4πsinθ/λ, where λ is the wavelength of the incident neutrons, and θ is half of the scattering angle. A mean neutron wavelength of 0.47 nm and 1.175 nm with 0.2 FWHM was produced by a multidisk mechanical velocity selector. Sample-detector distances 1.57 m and 5.50 m were used. By changing the wavelength and sample-detector distance, a *q* range of 0.08–2.5 nm^−1^ has been covered. In order to enhance the scattering contrast between hard and soft domains, all the samples were swollen by deuterated toluene for 48 h before measurements, and were measured in the swollen state. Immediately before the measurements, the swollen samples were transferred into quartz cells of path length 2 mm and ﬁlled with deuterated toluene. The schematic diagram of the setup can be found in the reference [16]. The acquisition times were about 30 min for each sample at each detector position. The scattering data were processed using BerSANS software [17]. The data reduction corrects the raw measured data for the contributions of the background, transmission, and scattering from empty cell. All the data were fitted by SASfit software [18], and the *q*-resolution has been taken into account by convoluting the theoretical curve with the instrument resolution function.

FTIR was performed using NICOLET 6700 FTIR spectrometer (Thermo Electron Scientific Instruments Corporation, Madison, WI, USA) in single attenuated total reflection mode on ZnSe crystal. Sixteen scans with spectral resolution 1 cm^−1^ were summed to achieve a good signal-to-noise ratio.

The GPC measurements were performed using a Biospher GMB 100 column (Labio, Prague, Czech Republic) filled with 10 μm sorbent particles. Tetrahydrofuran (Sigma-Aldrich, St. Louis, MO, USA), distilled and dried over molecular sieves (4 Å), was used as a mobile phase at a flow rate 1 mL/min. Small amount of toluene (2% (*w*/*v*)) was used as internal standard. The data from a refractive index detector were collected and treated by using CSW 1.7 software (Data Apex, Prague, Czech Republic). For the determination of molar masses, a universal calibration equation calculated from the data on polystyrene standards (Polymer Standards Service, Mainz, Germany) was used.

Calorimetric measurements were carried out using a DSC8500 apparatus (PerkinElmer, Waltham, MA, USA). Purge nitrogen was let through the DSC cell with a flow rate of 20 mL/min. The temperature of the equipment was calibrated with mercury, water and indium. The melting heat of indium was used for calibrating the heat flow. The samples were subjected to a cooling scan from 20 to −60 °C, holding at −60 °C for 5 min, followed by heating scan to 200 °C. Both scans were carried out at a rate of 10 °C/min. Glass transition temperatures were determined by Gaussian peak fitting to the numerical first derivative of the first heating thermogram.

## 3. Results

### 3.1. Small-Angle Neutron Scattering (SANS)

Polyurethanes containing 40 wt % or less HSs are known to have a discrete hard microdomain structure [10], which shows up as a broad interference peak in the small-angle scattering data. For a two phase system, it has been shown by Debye, Anderson, and Brumberger (DAB) that the correlation function can be expressed as exp (-*r*/*a*_cor_), where *a*_cor_ is the characteristic length over which the structural correlation decays [19]. The DAB model for random two-phase system takes the form

(1) $$P(q)=\frac{1}{{\left(1+a_{\text{cor}}{}^{2}q^{2}\right)}^{2}}$$

**Figure 2:**
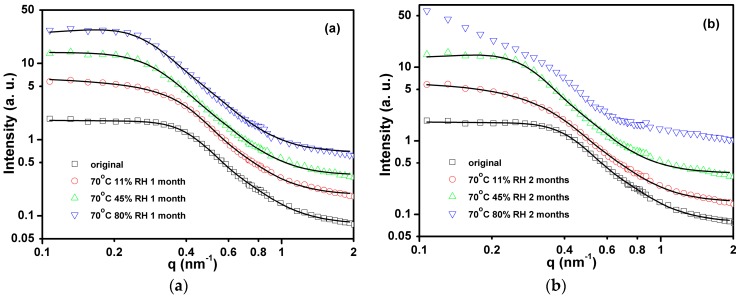
Small-angle neutron scattering (SANS) data of Estane aged in 11%, 45%, and 80% relative humidity (RH) environments at 70 °C for 1 (**a**) and 2 (**b**) months. The solid lines are fits by Debye, Anderson, and Brumberger (DAB)-Percus-Yevick (PY) model. The intensity profiles are shifted vertically.

**Figure 3:**
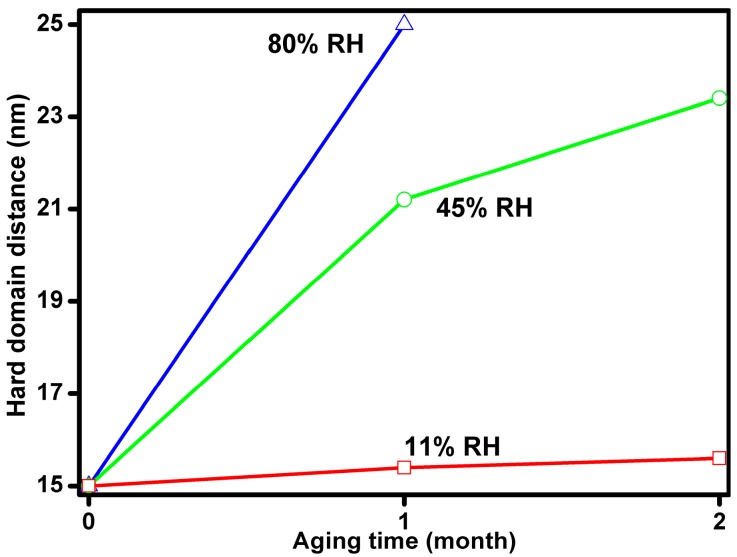
The hard domain distance (2*R*_HS_) variation with aging time and humidity at 70 °C.

In the present case, the *a*_cor_ can be considered as the hard domain size, since in Estane 5703 the HS content is low (23 wt %) and consequently the hard domains are much smaller than the chords crossing the soft and hard domains [11,20]. In this approach, the DAB model figures as an effective form factor of the irregularly shaped domains having smooth surfaces. The spatial arrangement of the domains can be approximated by the Percus-Yevick (PY) structure factor, applicable for hard sphere interaction, written as follows:

(2) $$S(q)=\frac{1}{1+24vG(A,v)/A}$$

in which *A* and *G* are algebraic functions of the *R*_HS_ and *v*, the hard sphere interaction radius and volume fraction, respectively. The first strong peak in the PY structure factor is related to the average distance between adjacent hard domains. Herein, we take the DAB combined with PY as an approximate empirical model to fit the scattering intensities by the equation

(3) $$I(q)=K\;\times \;P(q,a_{\text{cor}})\;\times \;S(q,R_{\text{HS}},v)\;+\;I_{B}$$

where *K* is a scale factor, and *I*_B_ is a constant which accounts for the incoherent scattering. The model performs well over a *q* range from 0.1 to 2 nm^−1^. Form factors of compact particle models, spherical and ellipsoidal, even taking into account the size distribution, resulted in worse fitting in the high *q* range, while required more free fitting parameters. Therefore, we consider that the present model is reasonable for describing the scattering data and following the development of the relevant structural parameters [16,21]. The *I*(*q*) curves of Estane aged in 11%, 45%, and 80% RH air at 70 °C for 1 and 2 months are shown in Figure 2. A broad peak is observed around *q* = 0.25 nm^−1^, which shifts towards smaller *q* with increasing aging time, indicating increase of the interdomain distance. Results of model fitting are shown in Table 1. Good agreement is found between the measured data and the model function, except for the sample aged at 70 °C for 2 months in 80% RH air. Within the studied aging time range, the higher humidity resulted in larger *a*_cor_ and *R*_HS_. After aging in 11% RH air for 1 month, the hard domain distance (2*R*_HS_) increases slightly and *a*_cor_ increases from 3.1 to 3.5 nm. After aging in 80% RH air for 1 month, 2*R*_HS_ increases from 14.9 to 24.9 nm and *a*_cor_ increases from 3.1 to 3.9 nm. The development of the hard domain distance with the aging time and humidity is presented in Figure 3. The increase rate of hard domain distance goes up with humidity. Continued aging at 80% RH for 2 months leads to a characteristic change of the scattering pattern: a strong intensity increase at small *q* values indicates the appearance of a new population of scatters or phase segregation on the length scale above 30 nm (estimated by π/q_min_). For this sample, the model used herein is no longer accurate due to the strong forward scattering, which masks the details of the domain structure.

### 3.2. Fourier Transform Infrared Spectroscopy (FTIR)

**Figure 4:**
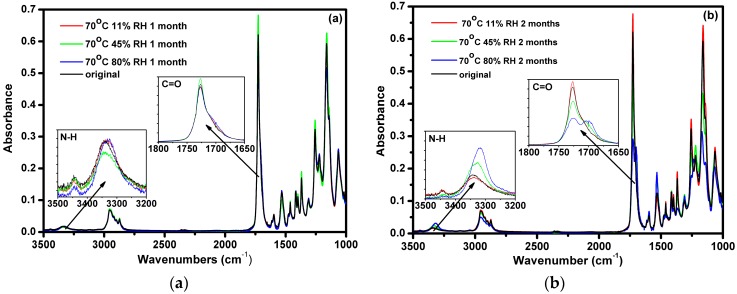
FTIR spectra of Estane aged at 70 °C in 11%, 45%, and 80% humid air for 1 month (**a**) and 2 months (**b**).

In segmented polyester urethanes, the H–bonded and free N–H and carbonyl C=O vibration provide the most useful information [21,22,23,24]. There are two types of H–bonds in Estane, *i.e.*, the inter-urethane and urethane-ester H–bonds. The absorption at about 3440 cm^−1^ corresponds to free N–H group, and the absorption band around 3340 cm^−1^ is assigned to H–bonded N–H group. After 1 month of aging, the IR data show no obvious variation on the N–H bands, only a slight shift to low frequency for the H–bonded N–H band (inset of Figure 4a). After 2 months of aging, the H–bonded N–H band of the sample exposed to 80% RH shifts to 3317 cm^−1^ at the expense of free N–H band (Figure 4b). The higher humidity and longer aging time induce higher absorbance of the H–bonded N–H band, implying a transition from free to H–bonded N–H groups.

The corresponding variation of the C=O stretching vibration band at 1760–1660 cm^−1^ is shown in the inset of Figure 4b. A shoulder appears at the right side (low frequency) of the strong absorbance peak (1726 cm^−1^). The absorbance of the shoulder increases with the humidity. For the sample aged in 80% RH for 2 months the C=O band splits into two peaks, located at 1726 and 1700 cm^−1^. The FTIR data of the aged samples reflect the packing changes of the polymer chains in hard and soft domains.

### 3.3. Gel Permeation Chromatography (GPC)

**Figure 5:**
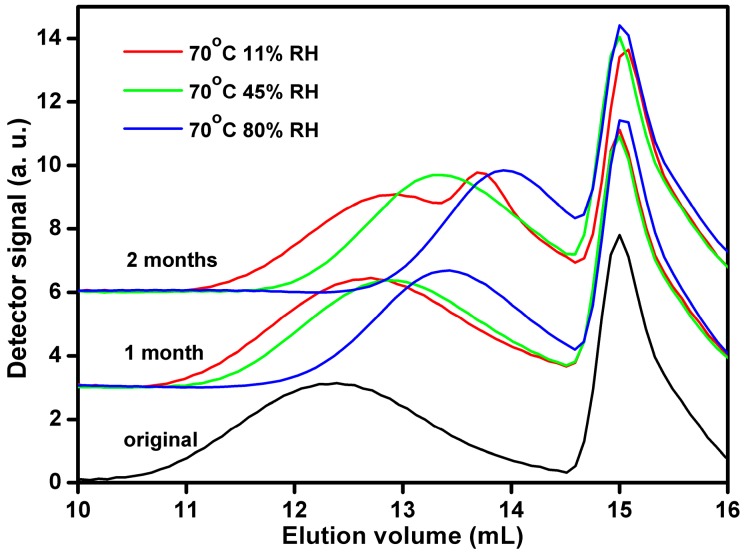
GPC curves for Estane aged at 70 °C in 11%, 45%, and 80% humid air for 1 and 2 months.

The original Estane sample exhibits a single broad GPC peak (Figure 5). Aging at 70 °C for 1 month causes a shift of the peak to higher elution volume, which indicates the shift of the molecular weight distribution (MWD) of the polymer chains to smaller molecular weights. The higher the RH is, the larger is the shift. The molecular weight decrease is stronger for Estane aged for 2 months. Interestingly, two GPC peaks appeared for the low RH (11%) sample. Obviously, MWD of the polymer chains becomes bimodal at these conditions. The position of the left side peak follows the overall trend of the continuous decrease of the averaged molecular weight of the aged samples (Table 2). The position of the right side peak corresponds to degradation products of much lower molecular weight centered at *ca.* 1500 g·mol^−1^. A probable reason of this effect is presence of a layer of more degraded polymer at the surface of specimen used for GPC analysis. The number- and weight-average molecular weight of the polymer (*M*n and *M*w) aged in 80% RH air for 2 months decreased by 20 times as compared with that of the original sample.

### 3.4. Differential Scanning Calorimetry (DSC)

**Figure 6:**
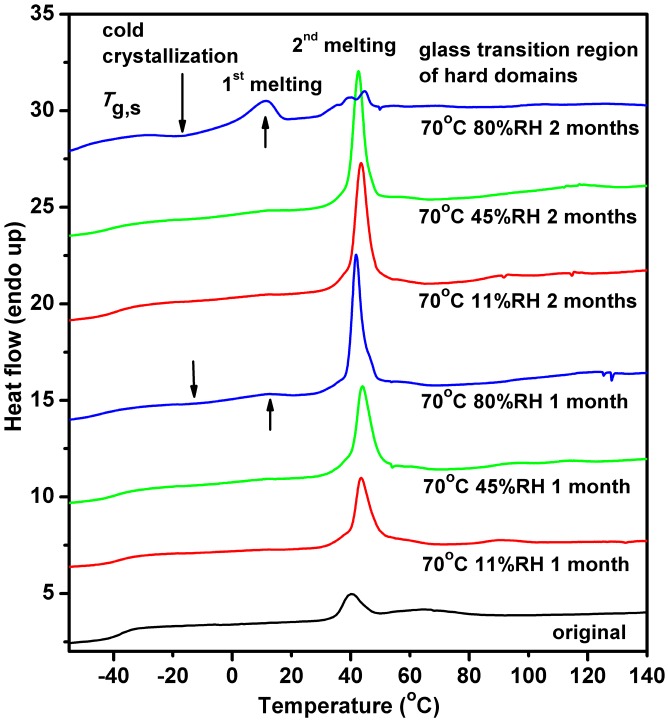
DSC thermograms of the Estane aged at 70 °C in 11%, 45%, and 80% humid air for 1 and 2 months during the first heating scan at a heating rate of 10 °C/min. The lines are shifted for clarity.

DSC thermograms for the original and aged Estane are shown in Figure 6. In heating of the original sample, the glass transition temperatures (*T*_g,s_) associated with the SSs is observed around −37.6 °C, followed by an endotherm with onset temperature (*T*_2_) at 33 °C. The *T*_g,s_ and *T*_2_ of the aged samples are lowered by a few degrees with increasing the RH and aging time relative to the original sample, except for the sample aged at the highest RH (*T*_g,s_ = −51 °C). The specific melting enthalpy (Δ*h*_2_) grows gradually, which means that the degree of crystallinity of the molecular chains is increased due to thermal and humid aging. Another wide and weak endotherm is present at 70–100 °C for all the samples, and the corresponding onset temperature (*T*_3_) shifts to higher temperature with aging. In the sample aged at the highest RH for 2 months two melting endothermic peaks emerge, centered at about 42 and 11 °C, and an exothermic peak centered at about −18 °C. This phenomenon is the same as the samples aged for 1 month at the highest RH, however, the changes are much less pronounced. The detailed parameters are listed in Table 3.

## 4. Discussion

### 4.1. The Phase Morphology of Aged Estane

The studied polyurethane, Estane 5703, has a weak scattering power for neutrons because of the modest contrast between the hard and soft domains. Therefore, we employed swelling by a selective solvent, deuterated toluene, to increase the contrast. This way, the coherent scattering signal increased by one order of magnitude and allowed the use of accurate model fitting to reveal details of the microphase morphology. The early work by Nierzwick *et al.* [25] proved that microphase separation influences the swelling properties of the material, and some information on the extent of microphase separation can be obtained from measuring the swelling ratio. When swelling is done with selective solvents, such as toluene, the hard domains are resistant to the solvent, and the domain structure can remain, though deformed, even in the swollen state. In the case of our samples, the deuterated toluene mainly swells the PBA SSs, and as a result, the interference peak of the swollen samples shifts towards lower *q*. In this experiment, we measured the structure change induced by aging, biased by the subsequent swelling of the material. Nevertheless, the trends of the domain size and interdomain distance variations are informative for the morphology development of the non-swollen aged materials.

**Figure 7:**
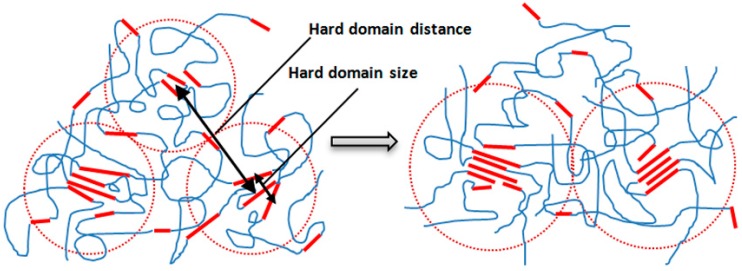
Schematic picture of the morphological development of the hard domains in aged Estane.

In our previous study [16], the *R*_HS_ remained unchanged for Estane aged at RT in 100% RH for 2 months. The present study provides additional information on the behavior of Estane treated at 70 °C at different humidities. The structural changes can be explained in the following way. The chemical structure of Estane has a compositional heterogeneity of the HSs length distribution. Upon aging, HSs of shorter lengths prefer to dissolve into the soft domains [10]. Accordingly, the number density of the hard domains, which is proportional to *v*/*R*_HS_^3^, is decreased for the aged Estane, while the domain distance increased, as shown in Table 1. Such behavior was also observed in annealed and thermal aged PUs with the same type of HSs [26,27]. Our SANS results illustrate that the increase of hard domain distance is strongly related to the influence of the environmental humidity (Figure 3). Considering the increase of hard domain size (*a*_cor_) and their distance (2*R*_HS_), it follows that a certain number of smaller hard domains disappear, being dissolved in the SSs matrix, while some of the larger hard domains grow even larger during the aging, as shown in Figure 7.

Decrease of hard domains in annealed waterborne polyurethanes has been also seen in TEM investigations employing ruthenium staining [28]. The domain size decrease after rearrangement of hard and soft domains by annealing at 80 °C had been estimated as about 1 nm. No chemical changes were considered to take place at this temperature, and the domain rearrangement was regarded as purely physical process, in contrast to the polyurethane studied in the present work for which the hydrolytic chain scission increased the mobility and promoted the morphological changes.

GPC data show that the molecular weight distribution of the polymer chains is progressively shifted to lower molecular weights with increasing aging time and humidity (Table 2). Compared to urethane groups, the ester group is more susceptible to hydrolysis, which reverts RCOOR' to polymeric acid (RCOOH) and polymeric alcohol (R'OH) [29,30,31]. The chain scission reaction takes place in the soft segments (SSs) and results in the decrease of the *T*_g,s_ (Table 2). Hence, the hard segments (HSs) and SSs mobility in the hydrolyzed samples is increased due to the reduction of the steric resistance. Consequently, the migration of the HSs from mixed phase or merging of some hard domains becomes favorable. Higher humidity results in the lower molecular weight and higher chain mobility, making easier the transformation towards fewer and larger hard domains.

### 4.2. The Hydrogen Bonds and Thermal Behavior of Aged Estane

Returning to the inset of the Figure 3b, the C=O bands splitting at 1726 and 1700 cm^−1^ of the sample aged in 80% RH for 2 months could be assigned to the H-bonded ester and H-bonded urethane bands, respectively [31]. It means that the number of inter-urethane H-bonds increases significantly, accompanied by the decrease of the urethane-ester H–bonds. This change is driven by the decrease of the molecular weight and enhanced mobility of the polymer chains, facilitating to the packing of the HSs to form H–bonds. Besides the changes of the hydrogen bonding interactions, the IR data also give information on the chemical change of the polymer. The decrease of the band at 1256 cm^−1^ corresponding to the C–O–C stretching vibration of the ester with increasing the humidity demonstrates that the hydrolytic chain scission reaction occurs at this functional group.

The glass transition temperature (*T*_g,h_) for the HSs (MDI+BDO) is around 75 °C [32], therefore the changes of the weak endotherm at 70–100 °C are related to the glass transition of the amorphous hard domains and dissolution of shorter length HSs. Larger hard domains possess higher *T*_g,h_, and the thermal and humid aging results in the increase of the hard domain size as observed by SANS, hence the *T*_3_ increases with aging. During the second heating, there is no endothermic peak at around 40 °C, indicating that longer time is necessary for ordering of the molecular chains; previously the samples were stored at RT for about one year. This strongest endotherm can be assigned to the melting of crystallites of well-ordered SSs. The rate of crystallization during the RT storage is strongly dependent on the mobility of the polymer repeating units. Decrease of the molecular weight due to hydrolysis and the subsequent increase of SSs mobility during the aging lead to faster crystallization during storage. Therefore, the degree of crystallinity of SSs is increased upon aging.

In summary, the above findings prove that, with decreased molecular weight, organization of HSs into domains is promoted by reduced steric hindrance in aged Estane, which appears to be the main mechanism of the morphological changes observed by SANS. While it has been reported earlier that Estane containing deuterated plasticizer underoges stronger phase segregation upon aging [8,9], new information was provided here for the behavior of pure Estane during thermal and humid aging by SANS model fitting and other techniques. Furthermore, in spite of the increase of the degree of phase separation, it can be inferred that the morphology development in aged Estane would lead to the deterioration of mechanical properties due to the reduction of the number density of hard domains, which act here as reinforcing fillers.

## 5. Conclusions

The microphase morphology and chemical modifications of Estane 5703 aged at three different humidities at 70 °C for 1 and 2 months have been studied by multiple techniques. The scattering data showed a significant increase of the domain distances with humidity and aging time. For the sample aged in high humidity for 2 months, the number of inter-urethane H-bonds increased significantly, accompanied by the decrease of the urethane-ester H-bonds. It is consistent with the increase of hard domain sizes and the degree of phase separation as measured by SANS. GPC results showed progressive shortening of the polyurethane chains with increasing humidity and aging time. The decrease of molecular weight of the polyurethane chains caused a decrease of the *T*_g,s_, leading to higher degree of crystallinity of the SSs after long-time storage of the samples at RT following the aging. The FTIR, GPC, and DSC results all indicated, from different perspectives, that the hydrolysis of ester links on polymer backbone at 70 °C is the main reason for the structural changes during aging at humid conditions.

## Figures and Tables

**Table 1:** Structural parameters obtained from SANS data on aged Estane 5703 samples swollen in toluene.

Samples	*R*_HS_ (nm)	*a*_cor_ (nm)	*v*	*v*/*R*_HS_^3^ (nm^−3^)
original	7.46 ± 0.05	3.07 ± 0.02	0.165 ± 0.003	4.0 × 10^−4^
70 °C 11% RH 1 month	7.69 ± 0.07	3.52 ± 0.03	0.136 ± 0.003	3.0 × 10^−4^
70 °C 45% RH 1 month	10.58 ± 0.19	3.71 ± 0.02	0.128 ± 0.006	1.1 × 10^−4^
70 °C 80% RH 1 month	12.46 ± 0.21	3.87 ± 0.02	0.173 ± 0.008	0.9 × 10^−4^
70 °C 11% RH 2months	7.82 ± 0.14	3.66 ± 0.07	0.098 ± 0.005	2.0 × 10^−4^
70 °C 45% RH 2 months	11.70 ± 0.11	4.65 ± 0.04	0.184 ± 0.007	1.0 × 10^−4^
70 °C 80% RH 2 months	–	–	–	

**Table 2:** Molecular weight distribution and thermal parameters obtained from gel permeation chromatography (GPC) data on aged Estane 5703 samples. *M*_n_ and *M*_w_ denote number- and weight-average (polystyrene equivalent) of molecular weight.

Sample	*M*_n_ (g/mol)	*M*_w_ (g/mol)	*M*_w_/*M*_n_
Original	33,000	120,000	3.6
70 °C 11% RH 1month	17,200	58,000	3.4
70 °C 45% RH 1month	11,000	34,000	3.1
70 °C 80% RH 1month	3,400	13,200	3.9
70 °C 11% RH 2months	9,500	36,000	3.8
70 °C 45% RH 2months	4,400	10,400	2.4
70 °C 80% RH 2months	1,750	2,500	1.4

**Table 3:** Thermal characteristics of aged Estane 5703 samples: glass transition temperature, *T*_g,s_, onset temperature of exotherm, *T*_exo_, onset temperatures, *T*_i_, and specific enthalpies, Δ*h*_i_ (i = 1,2,3), of endotherms.

Samples	*T*_g,s_ (°C)	*T*_exo_ (°C)	*T*_1_ (°C)	Δ*h*_1_ (J/g)	*T*_2_ (°C)	Δ*h*_2_ (J/g)	*T*_3_ (°C)	Δ*h*_3_ (J/g)
original	−37.6	-	-	-	33	6	-	9
70 °C 11% RH 1 month	−38.1	-	-	-	32	20	80	1.4
70 °C 45% RH 1 month	−39.1	-	-	-	30	23	82	2.2
70 °C 80% RH 1 month	−39.2	−20	−5	1.3	28	29	90	4.7
70 °C 11% RH 2 months	−41.4	-	-	-	30	24	75	2.8
70 °C 45% RH 2 months	−44.1	-	-	-	30	28	82	4.5
70 °C 80% RH 2 months	−51.0	−30	−5	10	28	20	90	8.1

## References

[1] Prisacariu C. (2011). Polyurethane Elastomers: From Morphology to Mechanical Aspects.

[2] Orler E.B., Wrobleski D.A., Smith M.E. Hydrolytic degradation of Estane 5703. Proceedings of the 22nd Aging, Compatibility and Stockpile Stewardship Conference.

[3] Orler E.B., Wrobleski D.A., Cooke D.W., Bennett B.L., Smith M.E., Jahan M.S. *Thermal Aging of Nitroplasticized Estane 5703*. In Proceedings of the 24th Aging, Compatibility and Stockpile Stewardship Conference.

[4] Salazar M.R., Lightfoot J.M., Russell B.G., Rodin W.A., McCarty M., Wrobleski D.A., Orler E.B., Spieker D.A., Assink R.A., Pack R.T. (2003). Degradation of a poly(ester urethane) Elastomer. III. Estane 5703 Hydrolysis: Experiments and Modeling. J. Polym. Sci. Part A Polym. Chem..

[5] Pierpoint S., Silverman J., Al-Sheikhly M. (2001). Effects of ionizing radiation on the aging of polyester based polyurethane binder. Radiat. Phys. Chem..

[6] Fambri L., Penati A., Kolarik J. (1995). Synthesis and hydrolytic stability of model poly(ester urethane ureas). Angew. Makromol. Chem..

[7] Thompson D.G., Osborn J.C., Kober E.M., Schoonover J.R. (2006). Effects of hydrolysis-induced molecular weight changes on the phase separation of a polyester polyurethane. Polym. Degrad. Stab..

[8] Mang J.T., Peterson P.D., Orler E.B., Wrobleski D.A., Langlois D.A., Espada L.I., Hjelm R.P. (2003). Small-angle neutron scattering study of a thermally aged, segmented poly(ester urethane) binder. Neutron News.

[9] Espada L.I., Mang J.T., Orler E.B., Wrobleski D.A., Langlois D.A., Hjelm R.P. (2001). Structural characterization of segmented polyurethanes by small angle neutron scattering. MRS Proc..

[10] Koberstein J.T., Galambos A.F., Leung L.M. (1992). Compression-molded polyurethane block copolymers. 1. Microdomain morphology and thermomechanical properties. Macromolecules.

[11] Krakovský I., Bubenikova Z., Urakawa H., Kajiwara K. (1997). Inhomogeneous structure of polyurethane networks based on poly(butadiene)diol: 1. The effect of the poly(butadiene) diol content. Polymer.

[12] Krakovský I., Urakawa H., Kajiwara K. (1997). Inhomogeneous structure of polyurethane networks based on poly (butadiene) diol: 2. Time-resolved SAXS study of the microphase separation. Polymer.

[13] Koberstein J.T., Russell T.P. (1986). Simultaneous SAXS-DSC study of multiple endothermic behavior in polyether-based polyurethane block copolymers. Macromolecules.

[14] Blundell D.J., Eeckhaut G., Fuller W., Mahendrasingam A., Martin C. (2002). Real time SAXS/stress–strain studies of thermoplastic polyurethanes at large strains. Polymer.

[15] Mang J.T., Hjelm R.P., Orler E.B., Wrobleski D.A. (2008). Small-angle neutron scattering of a solvent-swollen segmented polyurethane as a probe of solvent distribution and polymer domain composition. Macromolecules.

[16] Tian Q., Almásy L., Yan G., Sun G., Zhou X., Liu J., Rosta L., Chen B. (2014). Small-angle neutron scattering investigation of polyurethane aged in dry and wet air. Express Polym. Lett..

[17] Keiderling U. (2002). The new “BerSANS-PC” software for reduction and treatment of small angle neutron scattering data. Appl. Phys. A.

[18] Breßler I., Kohlbrecher J., Thünemann A.F. (2015). SASfit: A tool for small-angle scattering data analysis using a library of analytical expressions. J. Appl. Cryst..

[19] Debye P., Anderson R., Brumberger H. (1957). Scattering by an inhomogeneous solid. II. The correlation function and its application. J. Appl. Phys..

[20] Linliu K., Chen S.A., Yu T.L., Lin T.L., Lee C.H., Kai J.J., Chang S.L., Lin J.S. (1995). A small-angle X-ray scattering study of microphase separation transition of polyurethanes: Effect of hard segments. J. Polym. Res..

[21] Tian Q., Takács E., Krakovský I., Horváth Z.E., Rosta L., Almásy L. (2015). Study on the microstructure of polyester polyurethane irradiated in air and water. Polymers.

[22] Coleman M.M., Lee H.H., Skrovanek D.J., Painter P.C. (1986). Hydrogen bonding in polymers. 4. Infrared temperature studies of a simple polyurethane. Macromolecules.

[23] Coleman M.M., Skrovanek D., Hu J., Painter P.C. (1988). Hydrogen bonding in polymer blends. 1. FTIR studies of urethane-ether blends. Macromolecules.

[24] Luo N., Wang D.N., Ying S.K. (1997). Hydrogen-Bonding properties of segmented polyether poly(urethane urea) copolymer. Macromolecules.

[25] Nierzwicki W., Majewska Z. (1979). Swelling properties of urethane elastomers and their bearing on microphase separation. J. Appl. Polym. Sci..

[26] Yanagihara Y., Osak N., Murayama S., Saito H. (2013). Thermal annealing behavior and structure development of crystalline hard segment domain in a melt-quenched thermoplastic polyurethane. Polymer.

[27] Li X., Stribeck A., Schulz I., Pöselt E., Eling B., Hoell A. (2015). Nanostructure of thermally aged thermoplastic polyurethane and its evolution under strain. Eur. Polym. J..

[28] Princi E., Vicini S., Stagnaro P., Conzatti L. (2011). The nanostructured morphology of linear polyurethanes observed by transmission electron microscopy. Micron.

[29] Gardner R.J., Martin J.R. (1980). Effect of relative humidity on the mechanical properties of poly (1,4-butylene terephthalate). J. Appl. Polym. Sci..

[30] Vermette P., Griesser H.J., Laroche G., Guidoin R. (2001). Biomedical Applications of Polyurethanes.

[31] Schoonover J.R., Thompson D.G., Osborn J.C., Orler E.B., Wrobleski D.A., Marsh A.L., Wang H., Ralmer R.A. (2001). Infrared linear dichroism study of a hydrolytically degraded poly(ester urethane). Polym. Degrad. Stab..

[32] Li Y., Gao T., Liu J., Linliu K., Desper C.R., Chu B. (1992). Multiphase structure of a segmented polyurethane: Effects of temperature and annealing. Macromolecules.

